# Chronic Immune-Mediated Orchitis Is the Major Cause of Acquired Non-obstructive Azoospermia in Dogs

**DOI:** 10.3389/fvets.2022.865967

**Published:** 2022-04-01

**Authors:** Sandra Goericke-Pesch, Larena Reifarth, Christina Behrens Mathiesen, Gerhard Schuler, Anne-Kathrin Umbach, Hanna Körber

**Affiliations:** ^1^Department of Veterinary Sciences, Section for Veterinary Reproduction and Obstetrics, Faculty of Health and Medical Sciences, University of Copenhagen, Tåstrup, Denmark; ^2^Reproductive Unit – Clinic for Small Animals, University of Veterinary Medicine Hannover, Foundation, Hannover, Germany; ^3^Clinic for Obstetrics, Gynecology and Andrology of Large and Small Animals, Giessen, Germany; ^4^Veterinary Clinic for Small Animals Kaiserberg, Duisburg, Germany

**Keywords:** infertility, non-obstructive azoospermia (NOA), obstructive azoospermia (OA), autoimmune orchitis, hypothyroidism, thyroiditis, immune-cell infiltration

## Abstract

Azoospermia, the lack of spermatozoa in the ejaculate, is the most common finding in infertile but otherwise healthy male dogs and represents an increasing reproductive health issue in men, too. The diagnosis can be further classified as non-obstructive azoospermia and obstructive azoospermia due to an obstruction of the deferent ducts. Although non-obstructive azoospermia comprises more than half of azoospermic cases in men and is a common cause of infertility in the male dog, knowledge of the underlying etiology and pathophysiology is still strongly limited, and much uncertainty exists about the true incidence and possible treatment options. Therefore, this study aims to investigate and characterize infertile canine patients in detail by combining results of andrological examinations (clinical parameters, semen analysis, bacterial examination of semen, and *Brucella canis* serology), endocrine analysis (luteinizing hormone, testosterone, estradiol-17ß, and thyroid function), analysis of the alkaline phosphatase in seminal plasma, and histological assessment of testicular biopsies of 10 azoospermic dogs. Our results not only verify non-obstructive etiology for 9/10 cases of canine azoospermia but also further identified significant histopathological changes of the testicular tissue with severely disrupted spermatogenesis, including fibrotic remodeling, vacuolization, Sertoli-cell-only syndrome, tubular shadows, and an increase of the interstitial and vascular area. In addition, three dogs showed local and six dogs generalized immune-cell infiltration, indicating chronic immune-mediated orchitis. Only in one case (no. 1) that no immune cells were found, and obstructive azoospermia was suspected due to low alkaline phosphatase activity. Furthermore, the detection of anti-thyroideal antibodies in two dogs indicates an autoimmune thyroid disease and a correlation between the occurrence of thyroidal disorders and azoospermia. Our results confirm previous findings and contribute additional evidence suggesting that chronic immune-mediated orchitis is the major cause of infertility in dogs. Further studies should focus on uncovering underlying inflammatory processes behind spermatogenic failure in these cases and identify possible treatment options to (re-)initialize spermatogenesis.

## Introduction

Infertility is a global public health issue according to the World Health Organization (Geneva, Switzerland) (1) that affects not less than 186 million people worldwide—with male infertility contributing to more than half of all cases of global childlessness (2). Besides, there is sufficient evidence that male fertility or reproductive health has significantly declined over the last 40 to 60 years (3). Similarly to humans, infertility in dogs is a common (4–8) and increasing problem (9–11). Evaluation of the predominant causes showed similar results for both species.

Male dogs presented with infertility (“empty bitches”) at a veterinary reproduction specialist will undergo not only proper and careful andrological examination, including inspection, palpation, and possibly ultrasound examination of the genital organs, but also semen collection and semen analysis (6, 12–14). Thus far, the main analyses used to evaluate semen in domestic animals are divided into qualitative (semen volume, aspect, consistency, and pH) and quantitative parameters (sperm concentration/count, motility, morphology, vitality, DNA fragmentation, and morphometry) (15). Although assumed to be of male origin, “infertility” can be associated with the wrong timing of mating or even infertility in the bitch. Nevertheless, losing a successful male dog for further breeding is often associated with high emotional pressure and even substantial financial losses for the owner (16).

The most common finding in infertile but otherwise healthy male dogs is azoospermia, the lack of spermatozoa in the ejaculate (17). With a prevalence of up to 34.8%, it is considered to be one of the main reasons for reproductive problems in the male dog and can be identified in 15% of infertile men, too (18). The diagnosis should be confirmed by the absence of spermatozoa in urinalysis (13). However, underlying causes for infertility and azoospermia might be multifactorial. Azoospermia can further be differentiated into non-obstructive azoospermia (NOA), also termed “true azoospermia,” and obstructive azoospermia (OA), due to an obstruction of the deferent ducts. Although OA comprises 40% of azoospermic cases in men (18), only two references describe OA in dogs (19, 20). Not only regarding published literature, but also according to our own clinical experiences, OA is rare, and NOA is the most common cause of azoospermia in dogs (21). In men, approximately 1% of the male population and 10% of all infertile male patients are affected by NOA (1). To differentiate between OA und NOA, determination of the alkaline phosphatase (AlP) in the seminal plasma is used in various species, including the dog, as this enzyme is highly expressed in the epididymal tail [men: (22); stallion: (23, 24); dog: (13, 21, 25–27)]. Although NOA is easy to diagnose after adequate, preferably repeated semen collections, the confirmed absence of spermatozoa in the ejaculate and determination of the AlP in seminal plasma, identifying the underlying etiology or cause for azoospermia requires further diagnostics.

Diagnostics should include testing for genetic abnormalities and other possible pretesticular and testicular causes of NOA. Inherited genetic abnormalities, including microdeletions of the Y chromosome, abnormal karyotypes, and missense mutations of “fertility” genes, are known to be causative in 25% of human NOA cases (1, 28). However, only little is known about the impact of genetic factors and the heritability of azoospermia in the dog. Pretesticular causes of NOA also include pituitary or thyroidal dysfunction, such as primary or secondary pituitary hypogonadism [men: (18, 29), dog: (30)], Cushing's disease [men: (31); dog: (16, 30)], or hypothyroidism [men: (32–35); dog: (16, 30, 36)] with the latter a postulated but not confirmed cause for male canine infertility (37, 38). Whether endocrinopathies or disturbances of the hypothalamic–pituitary–gonadal axis have a direct or an indirect effect on fertility *via* testosterone production is controversially discussed (39–43). Clinically relevant testicular causes of NOA are variable and include bilateral cryptorchidism (however, not in breeding studs), Sertoli-cell-only syndrome, and disturbed/disrupted spermatogenesis due to testicular trauma, neoplasia, and/or orchitis/epididymitis (17). Whereas, neoplasia and acute infectious orchitis/epididymitis can be easily diagnosed by clinical examination, sonography, and bacteriological examination of a semen sample (orchitis/epididymitis), clinical history provides insights into testicular trauma or irradiation, and exploration of other underlying causes of NOA, such as autoimmune/immune-mediated orchitis, might be challenging and requires histological assessment of testicular tissues, either obtained by castration or by testis biopsy. Without a doubt, identification of the cause is desirable—also for identification of possible adequate therapeutic strategies.

Consequently, this study aimed to investigate infertile azoospermic dogs in detail and combine results of endocrine analysis [luteinizing hormone (LH), testosterone, estradiol-17ß, and thyroid function] with those of clinical andrological parameters, semen analysis, serology for *Brucella canis*, bacteriological examination of the semen sample for aerobic culture, determination of the AlP from seminal plasma, and testicular histology obtained from bilateral testis biopsies.

## Materials and Methods

### Study Design

**Figure 1:**
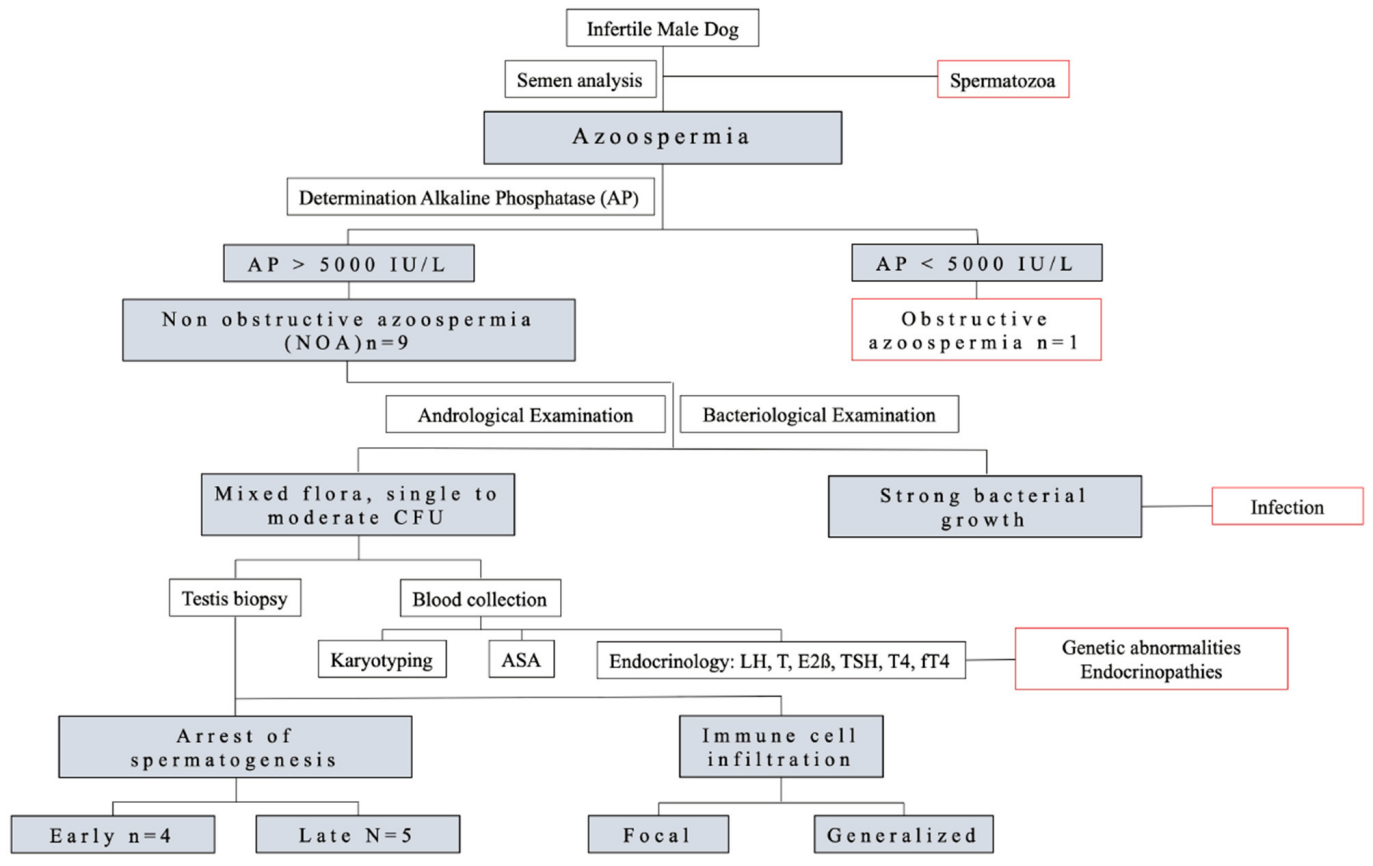
Flowchart of the experimental design.

Infertile male dogs with irreversible azoospermia were included in the study. A brief flowchart of the clinical procedures is given in Figure 1. Testicular biopsies have been collected for diagnostic purposes after the written owner's consent.

### Animals

Ten infertile but clinically healthy male dogs were included after confirmation of permanent, irreversible azoospermia (repeatedly confirmed). Semen collections had been performed at least twice with an interval of at least 3 months. Dogs had a mean age of 6.8 ± 2.0 (range: 4.7–10.1) years and belonged to nine different breeds. Breeds of the dogs included Danish Spitz, Smooth Collie (*n* = 2; father and son), Rough Collie, Cairn Terrier, Miniature Poodle, Labrador Retriever, Icelandic Sheepdog, Welsh Corgi Pembroke, and Coton de Tuléar. A detailed reproductive history, including breeding management (age at first mating, number of matings, time point of last mating, and last successful mating—if any), number of mated/pregnant bitches (and if breeding management was performed in mated bitches), fertility (puppies/litter), mating behavior including libido, and mating itself (“normal” with tie, etc.), was obtained.

### Clinical and Andrological Examination

Clinical physical examinations were performed after semen collection and before sedation of sampling of testicular biopsies. Besides, an andrological examination, including measurement of testicular dimensions (length × height × width) with a caliper, was performed as previously described (44, 45), as well as an ultrasound examination to evaluate the testes and prostate.

### Semen and Seminal Plasma Analysis

Semen samples were collected in the presence of an estrous teaser bitch, as previously reported (46, 47). Semen analysis included a macroscopical examination (volume, color, consistency, and odor), a chemical–physical examination (pH value using pH indicator paper, Merck, Darmstadt, Germany), and a microscopical examination for the presence of spermatozoa. An aliquot of the semen sample was sent for aerobic bacteriological analysis to the Department of Veterinary Disease Biology at the Faculty of Health and Medical Sciences, University of Copenhagen, Denmark, and processed according to routine diagnostic procedures. Identification of bacterial species was made by matrix-assisted laser desorption/ionization-time of flight (MALDI-TOF). Additionally, the number of colony-forming units (CFU) was recorded.

To confirm the absence of spermatozoa, the obtained fractions of the ejaculates were centrifuged at 700 × *g* for 10 min (Hettich Rotina® 380 R, Tuttlingen, Germany). Afterward, the supernatant was pipetted into an Eppendorf tube (Eppendorf, Germany), and the remaining cell pellet was microscopically rechecked and confirmed to be sperm-free.

The supernatant, the seminal plasma, of the second fraction was used for the analysis of the AlP as previously described (21, 47). Briefly, the second fraction of the semen sample was centrifuged twice, with the supernatant being further processed for analysis using EPAC-TM 5430 and EPAC 6410 (Eppendorf).

### Blood Collection and Hormone Analysis

Blood samples were collected from the cephalic vein into empty, ethylenediaminetetraacetic acid (EDTA)-containing, and heparinized tubes. EDTA samples were used for karyotyping. Serum and heparinized plasma were obtained after centrifugation and immediately frozen at −80°C for subsequent hormone analysis. Additionally, an aliquot of the serum was sent for *B. canis* antibody titer analysis to the Institute for Hygiene and Infectious Diseases, Justus-Liebig-University Gießen (Germany) (48).

LH was determined by an in-house heterologous competitive enzyme immunoassay (49). The lower limit of detection for LH was <0.2 ng/ml, and the intraassay coefficients of variation were between 6.9 and 15.9% and the interassay coefficients of variation between 6.7 and 11.4%. Testosterone and estradiol-17ß were determined by in-house radioimmunoassays as previously described (50, 51). For testosterone, the intra- and interassay coefficients of variation were between 7.8 and 9.0%. The lower limit of detection was <0.1 ng/ml. For estradiol, the lower limit of detection was <2 pg/ml, the intra- and interassay coefficients of variation were between 9.4 and 17.5%.

Thyroid hormones [thyroxine (T4) and canine thyroid-stimulating hormone (cTSH)] and anti-thyroideal antibodies were analyzed as previously described (52–54).

### Testicular Biopsies and Tissue Processing

Testicular scissors biopsies from the left and right testis of each azoospermic dog were collected under general anesthesia according to routine surgical procedures (55). Parenchyma samples of each side were cut into two pieces: A smaller piece was put into RNAlater® (Ambion Biotechnologie GmbH, Wiesbaden, Germany); the larger piece was fixed in Bouin's solution for 24 h at 4°C. Bouin-fixed samples were then washed several times in 70% ethanol, infiltrated with paraffin (60°C; overnight), embedded in paraffin wax, and mounted onto blocks as previously described (44). Twenty sections (2–3 μm) were cut and dried; the slides 1, 5, 10, 15, and 20 were hematoxylin–eosin-stained and mounted in Histokitt (Assistent, Osterode, Germany).

For comparative aspects regarding histology, testes of five normospermic dogs presented for routine castration served as controls. Semen collection and analysis were performed as described earlier, and semen parameters were found to be within the reference ranges (47, 56). Immediately after removal, the testes were cut longitudinally, and approximately 0.5-cm^3^ parenchyma samples were taken from the area between the tunica albuginea and the mediastinum testis. The tissue was processed exactly in the same way as the samples from the azoospermic patients.

### Histological Evaluation

The histological examination of the testicular tissue was performed using hematoxylin–eosin-stained slides. The evaluator was blinded at the time of evaluation to avoid any bias. In 50 approximately round tubules per testis (right/left) using a minimum of four sections, the stage of the arrest of spermatogenesis (most developed germ cell or presence of Sertoli-cell-only tubules) was evaluated. Additionally, the following histological parameters were scored semiquantitatively: thickening of the basal membrane (0 to ++++), presence of fibrosis (0 to +++), infiltration with immune cells (0: no, +: focal, ++: generalized infiltration), presence of a tubular lumen (absent/present), frequency of divisions (0 to ++), and presence of intratubular vacuolisation (0 to +++). The evaluation was done at 400-fold magnification.

Furthermore, a morphometric analysis of the three compartments, tubular, interstitial, and vascular compartments (with the latter containing larger blood and lymphatic vessels), was performed on both samples of azoospermic and normospermic patients. Ten random photographs of the histological slides of each testis were taken at 200-fold magnification. The software program University of Texas Health Science Center, San Antonio ImageTool version 3.0 (UTHSCSA) was used to calculate the percentage distribution f the tubular, interstitial, and vascular compartments as previously described (57, 58).

### Statistical Evaluation

Overall data were presented descriptively. Individual results were presented, but additionally, some data were summarized. Results were presented as mean ± standard deviation in the case of normal distribution (months at the time of inclusion, interstitial area, tubular area, and vascular area). Additionally, the range was presented for selected parameters as age at the time of inclusion, AlP activity, LH-, T-, and estradiol concentrations, T4, fT4, and cTSH.

In the case of morphometry, statistical calculations were performed comparing results obtained from dogs suffering from NOA to control dogs with normospermic ejaculates and “normal” spermatogenesis. Data were initially tested for normal distribution using Shapiro–Wilk test. As data were normally distributed, results per parameter of NOA and control dogs were analyzed using an unpaired *t*-test.

For statistical analysis, the statistical softwares R, version 3.2.2 (2015-08-14), and GraphPad Prism v. 7.02 (GraphPad software. Inc., San Diego, CA, USA) were used. Values were considered statistically significant at a level of *p* ≤ 0.05.

## Results

Individual results of each dog are presented in Table 1.

**Table 1:** Overview about results obtained from individual patients with permanent azoospermia including reproductive history of successful mating (yes/no), months since last successful mating (last mating), activity of alkaline phosphatase (AlP) [IU/l], result of aerobic bacterial culture and results of endocrine analysis: concentrations of luteinizing hormone (LH) [ng/ml], testosterone (T) [nmol/L], estradiol-17ß (E2ß) [pmol/L], thyroxine (T4) [μg/dl], canine thyroid-stimulating hormone (cTSH) [ng/ml], thyroglobulin autoantibodies (TgAA) [%]. Additionally, overall result of histological assessment—early or late arrest [arrest (early/late)] as well as infiltration with immune cells (IC: no immune cells [no], focal [focal], and generalized [gen.] immune cell infiltration—was recorded.

**No**	**Sired before**	**Last mating**	**AlP**	**Bacterial growth**	**LH**	**T**	**E2ß**	**T4**	**cTSH**	**TgAA**	**Arrest**	**IC**
		**[Months]**	**[IU/L]**		**[ng/ml]**	**[nmol/L]**	**[pmol/L]**	**[μg/dl]**	**[ng/ml]**	**[%]**		
1	No	*	100	*Staph. pseud-intermedius*	4.0	5.50	92.50	3.1	0.37	32.8	Late	No
2	No	*	83,700	-	1.0	3.8	106.1	1.1	0.83	<10	Early	Gen.
3	Yes	5	31,800	-	2.8	4.9	106.4	3.4	0.07	15.6	Late	Gen.
4	Yes	48	50,500	-	0.8	2.0	66.0	1.4	0.15	<10	Late	Local
5	Yes	42	22,400	-	0.5	2.2	116.4	0.93	0.55	<10	Late	Gen.
6	Yes	10	45,500	*Moraxella osloensis*	2.0	10.3	141.5	1.9	0.07	<10	Late	Local
7	Yes	36	72,900	*Pasteurella multocida*	1.4	6.8	113.1	1.9	0.07	<10	Early	Gen.
8	No	*	1,600^#^	*	2.8	9.3	117.2	2.4	0.03	<10	Early	Gen.
9	Yes	42	171,800	*Strept. canis, Staph. pseudintermedius*	1.9	1.8	99.3	0.7	0.14	<10	Early	Gen.
10	Yes	12	15,510	-	1.9	8.0	91.2	2.7	0.06	<10	Late	Local

# *Anxious, nervous during semen collection*.

*Staph.: Staphylococcus*.

*Strept.: Streptococcus*.

* *Not applicable (last mating)/no sample obtained (bacterial growth)*.

*^−^No bacterial growth detected*.

### Results of Reproductive History and Andrological Examination

In total, 7 of 10 dogs sired successfully before, with the last successful mating being 27.9 ± 18.1 (range: 5–48) months ago at the time of inclusion.

Regarding the results of the andrological examination, nine dogs had smaller testis (*n* = 5; 2, 7–10) or testis within the lower reference (*n* = 4; nos. 1, 3, 5, and 6) compared with what was expected for the respective sizes/body weights of the dogs (45). Regarding testicular consistency, both testes had the same consistency. The consistency was soft in 15 of 20 testes. One dog had a small prepuce and penis (no. 9). Ultrasound examination revealed a broad Rete testis associated with an inhomogeneous structure of the testicular parenchyma in most testes. Besides, ultrasound examination revealed signs of chronic prostatitis in one dog (no. 4).

Karyotyping was attempted in the three azoospermic dogs (two never sired and the one with the small prepuce and penis). All dogs were SRY positive; karyotyping succeeded in one and was XY.

### Alkaline Phosphatase Activity

The activity of the AlP ranged from 100 to 171,800 IU/L, with two dogs having total activities below 5,000 IU/L. Dog no. 1 (AlP = 100 IU/L) showed high libido and normal ejaculation reflexes during collection, indicating suspicion of OA. Different from this, dog no. 8 (AlP = 1,600 IU/L) was anxious and reluctant toward handling so that incomplete ejaculation could not be ruled out as a cause for relatively low AlP activity. However, in case of normospermia, at least some spermatozoa are found in ejaculates even with these low AlP activities (1,600 IU/L) according to own experiences resulting in the assumption of true azoospermia.

### Bacteriological Examination and *B. canis* Serology

Regarding aerobic bacterial culturing, one sample was excluded, as the dog had been treated with procaine penicillin the day before presentation in the clinic for other than fertility reasons. (This bacteriological sample was sterile) In the remaining nine samples, five samples were reported to be sterile in aerobic culture, whereas bacterial growth was identified in four samples (three times monoculture, once two bacterial species). Details on bacteria species identified are given in Table 1. For all results, the number of CFUs ranged between 600 and 2,000 CFU/ml.

Antibodies against *B. canis* were not detectable by serum tube agglutination test in any of the 10 samples (O.I.E. “manual of diagnostic tests and vaccines” for terrestrial animals, chapter 2.3.1. no. 3b), indicating that azoospermia was not related to canine brucellosis. Besides, none of the dogs had any clinical signs of *B. canis* infection.

### Endocrine Analysis

Detailed results of endocrine parameters are given in Table 1. LH concentrations ranged between 0.5 and 4.0 ng/ml. Testosterone concentrations ranged between 1.8 and 10.3 nmol/L. Estradiol concentrations varied between 66.0 and 141.5 pmol/L. Regarding thyroid hormones, two dogs (nos. 5 and 9) had decreased T4 levels (reference: 1.1–3.0 μg/dl); two dogs (nos. 2 and 5) had cTSH concentrations above the reference (reference: <0.45 ng/ml), indicating hypothyroidism in dog no. 5. Free T4 concentrations were within the reference range (1.2–2.4 ng/dl) for all dogs. Thyroglobulin autoantibodies were above the reference levels in two different dogs (nos. 1 and 3; reference: <10%).

### Histological Evaluation and Morphometry

**Figure 2:**
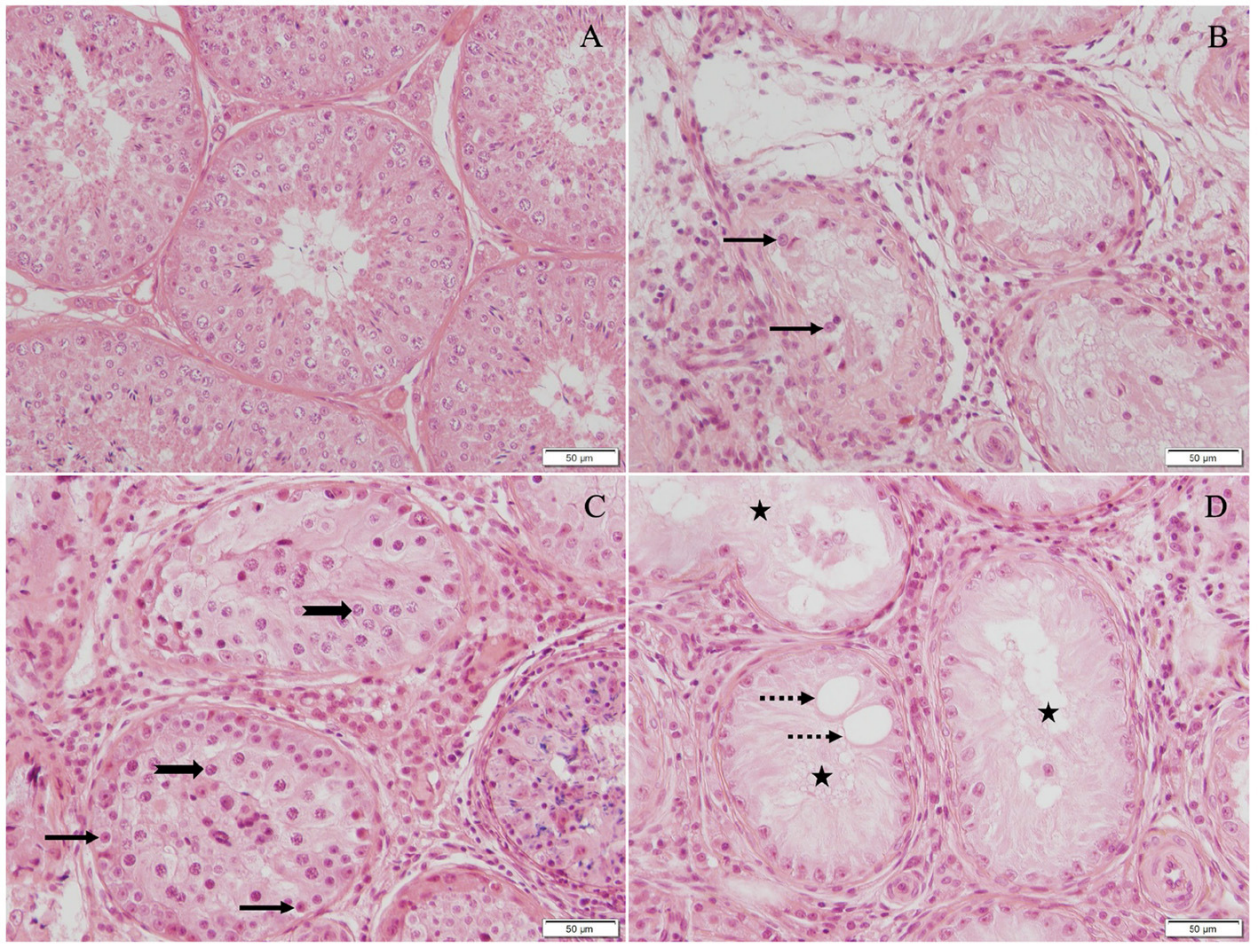
Haematoxylin–eosin-stained testicular tissue (200× magnification): **(A)** Normospermic dogs with unaltered spermatogenesis served as control group, **(B)** early arrest with germ cell development arrested at stage of spermatogonia (*black arrow*), **(C)** late arrest with germ cell development arrested at stage of spermatocytes (*thick black arrow*), round, elongating, or elongated spermatids, and **(D)** Sertoli-cell-only syndrome with germ cells completely lacking (*black star*), and vacuoles are present in seminiferous tubules (*dotted arrow*). Fibrosis and massive immune-cell infiltration indicate presence of immune-related orchitis in all cases of NOA **(B**–**D)**.

**Figure 3:**
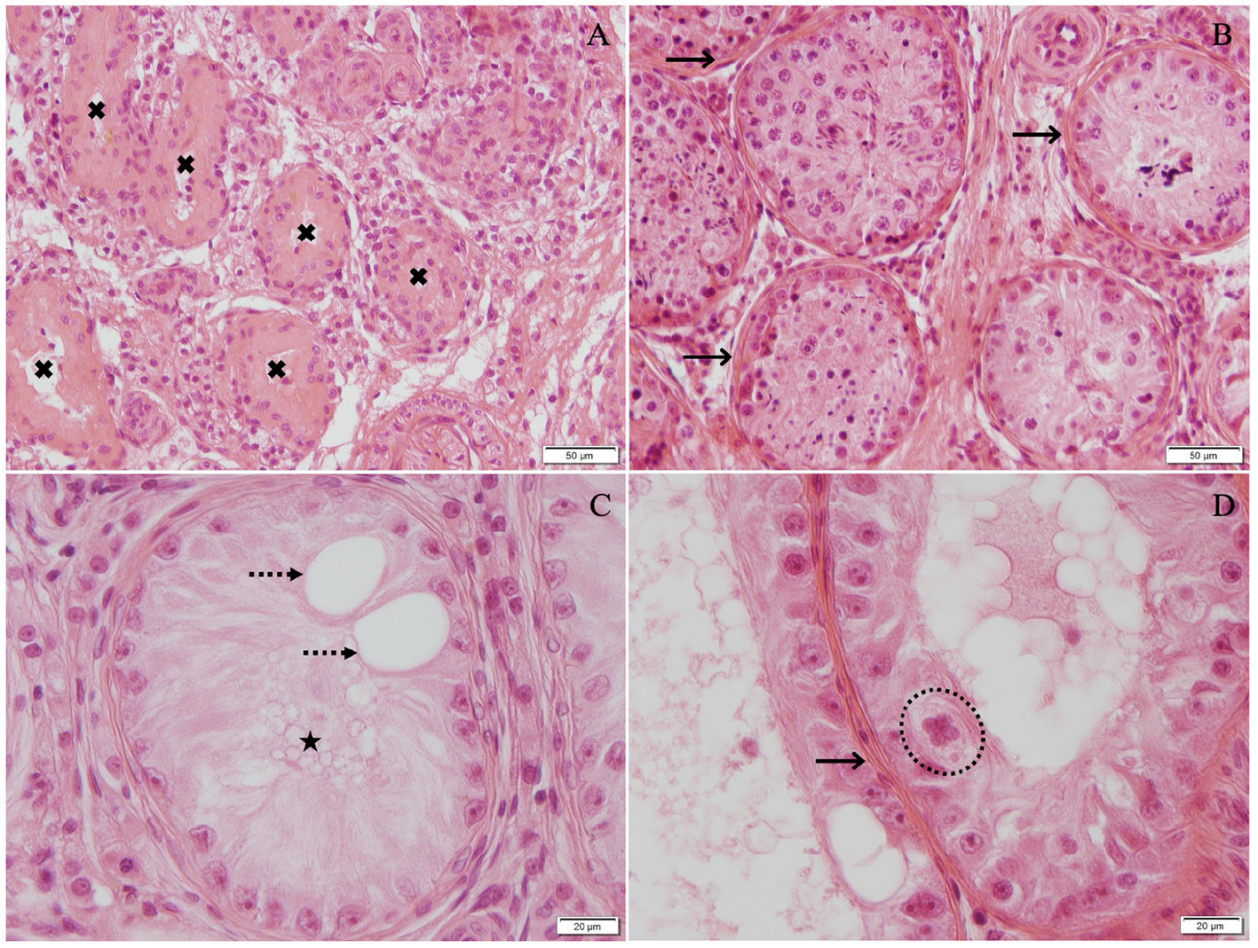
Hematoxylin–eosin-stained testicular tissue [**(A,B)** 200× magnification; **(C,D)** 400× magnification]: **(A)** Tubular shadows, tubules containing neither germ nor Sertoli cells, and basal membrane is very thickened (*black cross*), **(B)** late arrest with fibrosis thickened basal membrane (*black arrow*), **(C)** Sertoli-cell-only syndrome (*black star*) with vacuoles (*dotted arrow*), and **(D)** early arrest with thickened basal membrane (*black arrow*) and multinucleated germ cells (*dotted circle*).

All dogs suffering from azoospermia had significant changes in the testicular histology with severely disrupted spermatogenesis. The arrest of spermatogenesis was classified either as early or late arrest of spermatogenesis, according to Bergmann and Kliesch (55). The term “early arrest of spermatogenesis” was used when the majority of the round tubules counted in the biopsy were either arrested at the stage of spermatogonia (Figure 2B) or germ cells were completely lacking, so-called Sertoli-cell-only syndrome (Figure 2D). The term “late arrest of spermatogenesis” was used when the majority of the round tubules evaluated in the biopsy were arrested at the stage of spermatocytes, round spermatids, or elongating spermatids and in case single fully elongated spermatids were present (Figure 2C). Four of 10 dogs had “early arrest of spermatogenesis,” and 6 of 10 dogs had “late arrest of spermatogenesis” (Figure 2). All four dogs with “early arrest of spermatogenesis” and two additional dogs showed generalized histopathological changes of the testicular tissue with generalized immune-cell infiltration, indicating the presence of (auto)immune-related orchitis (Tables 1, 2). An additional three dogs had local immune-cell infiltrations. Only in one dog (no. 1) no immune cells were found. Interestingly, in this dog (no. 1), OA was suspected due to low AlP activity but high libido and normal reflexes during collection. In the case of dog no. 8, where incomplete ejaculation was considered to be the reason for low AlP activity, testicular histology was identical with the remaining eight cases indicating NOA, too. Further histological details, such as thickening of the basal membrane, fibrosis, and presence of vacuolization, were found and are given in Table 2 and shown in Figure 3. Besides and different to normospermic controls, seminiferous tubules in NOA dogs were lacking a tubular lumen as clearly visible in the assessed approximately round tubules (Figure 2C). Furthermore, no meiotic figures were grossly observed in any of the azoospermic dogs except for in no. 1. In all azoospermic dogs, some Sertoli cells were found to have a round or ovoid nucleus rather than the normal irregular shape, and these nuclei were not located at the basal membrane but located more adluminally, consistent with Sertoli-cell dysfunction as described earlier in the gonadotropin releasing hormone agonist downregulated implant canine testicular tissues (44, 59). This was not found in any of the testicular specimens of the control dogs (Figure 2A). Furthermore, some of the tubules (nearly all in no. 9) revealed tubular shadows, a phenomenon where the round tubules contain neither germ nor Sertoli cells, and the basal membrane is extremely thickened (55) (Figure 3A), indicating the final stage of (autoimmune) orchitis.

**Table 2:** Histological characterization of testicular biopsies obtained from azoospermic dogs. Table describes arrest of spermatogenesis in more detail by giving details on 1. which level majority of tubules are arrested on (arrest) and 2. most developed germ cell found. Besides presence of immune cells (immune cells: 0-++), thickening of basal membrane (basal membrane: 0-+++), interstitial fibrosis (fibrosis: 0-+++), and presence of intratubular vacuoles (vacuoles: 0-+++) are scored semiquantitatively.

**No**.	**Arrest**	**Most developed germ cell**	**Immune cells**	**Basal membrane**	**Interstitial fibrosis**	**Vacuoles**
1	rspt	espt	0	+	0	++
2	SCO	spg	++	+++	+++	+++
3	rspt	espt	++	+++	++	++
4	rspt	espt	+	++	0	+
5	rspt/spg	edspt/espt	++	+++	++	++
6	rspt	espt	+	++	+	+++
7	SCO	spc	++	+++	++	+++
8	SCO	spg	++	+++	++	+++
9	spg	rspt	++	+++	++	+++
10	rspt	espt	+	++	+	+

*Spermatogonia (spg), spermatocytes (spc), round spermatids (rspt), elongating spermatids (espt) or mainly tubules with Sertoli-cell-only (SCO), elongated spermatid (edspt)*.

**Figure 4:**
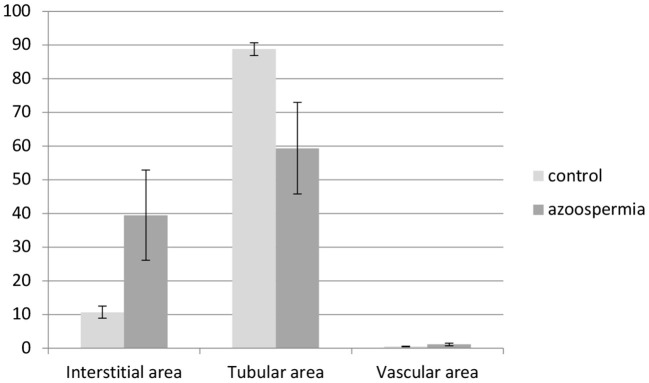
Morphometry of interstitial, tubular, and vascular areas (%) of testicular tissues obtained from azoospermic and normospermic dogs (control). Interstitial and vascular areas are significantly increased (*p* < 0.01 and *p* < 0.0001, respectively), whereas tubular area is significantly decreased in testicular tissues from azoospermic compared with normospermic dogs (*p* < 0.0001).

The morphometrical analysis did not reveal significant differences between the left and the right testes. Comparing, however, morphometrical results of testicular tissue of azoospermic dogs with that of normospermic dogs, the interstitial area was significantly increased (azoospermia: 39.51 ± 13.42%, control: 10.72 ± 0.40%, *p* < 0.01), whereas the tubular area was significantly reduced (azoospermia: 59.42 ± 13.60%, control: 88.81 ± 1.85%, *p* < 0.0001). Interestingly, the vascular area was also increased in case of azoospermia (azoospermia: 1.07 ± 0.40, control: 0.47 ± 0.08%, *p* < 0.0001) (Figure 4).

## Discussion

Similar to earlier research (13, 19–21, 30, 41), our results clearly confirm that NOA is a common cause of azoospermia in dogs. Nevertheless, this is the first study that investigates and characterizes azoospermia and its possible causes in infertile canine patients on the clinical, andrological, endocrinological, and histological levels.

### Affected Breeds, Reproductive History, and Andrological Examination

Various breeds (Beagle, Labrador Retriever, Scottish Terrier, Kerry Blue Terrier, English Cocker Spaniel, Weimaraner, German Shorthair Pointer, Pug, and Newfoundland) have been described earlier to be affected by NOA—mainly in case reports or smaller cohorts (19, 30, 41, 60–63). Our random study population includes even further breeds and is consequently in good agreement with clinical observations that dogs of all breeds might be affected by azoospermia and NOA. Although the Labrador Retriever seems to be overrepresented in the present literature (19, 41, 60, 64), we only included one Labrador Retriever, clearly emphasizing that large populations of infertile dogs are required to identify real breed predispositions for NOA. Considering that heritability was discussed earlier for infertility/NOA, it seems noteworthy that two related Smooth Collies (a sire and his son) were included in the present study. Six of 18 dogs with azoospermia or suspected azoospermia had male relatives with a confirmed reproductive disorder in one study (19), besides three of four infertile males in another study were related (64). Even if the number of cases with familiar infertility is low in the present study group, the observation of familiar acquired infertility might support the hypothesis that in- or line-breeding are a cause for azoospermia and negatively affect reproductive performance in various species [dog: (65, 66); cat: (67–69)]. Regarding clinical and andrological examination, 9 of 10 dogs had small-sized testis or testis within the lower reference described earlier required for “normospermia” (45). These results corroborate the findings of previous works that reported a decreased testicular volume (70) or small soft, atrophic testes (18) in cases of NOA or germ cell aplasia (29) in men. Further findings of the included dogs with soft testes, a small epididymal tail, inhomogeneous testicular parenchyma, and an enlarged and broadened Rete testis as identified by ultrasound examination are very unspecific but in good agreement with findings in men with NOA (70, 71). Although, in the vast majority of patients in men, NOA can be clinically distinguished from OA by a profound analysis of diagnostic parameters (including history, physical examination, and hormonal analysis) (71), analysis of the AlP in the seminal plasma is the key parameter to differentiate between OA und NOA in dogs as mentioned earlier (13, 21, 25–27). The reference values confirming the normal course of ejaculation reflexes and emptying of the epididymal tail vary, however, between studies. In contrast, Johnston et al. (17) consider AlP activities >5,000 IU/L indicative, Goericke-Pesch and Wehrend (21) describe that AlP activities >30,000 IU/L in the second fraction of the seminal plasma of an ejaculate confirm complete emptying of the epididymal tail in the dog. Low activities associated with azoospermia as found in dog nos. 1 and 8 can be related to obstruction or incomplete ejaculation reflexes. Consequently, the stud's behavior during semen collection should be carefully evaluated to possibly identify incomplete ejaculation. Besides, urine collection and analysis for presence or absence of spermatozoa is recommended to confirm the diagnosis: Spermatozoa are released into the bladder not only in regular intervals in healthy males but also during incomplete/retrograde ejaculation. Consequently, low seminal plasma AlP with no spermatozoa in the ejaculate but spermatozoa in the urine indicates incomplete ejaculation and/or retrograde ejaculation. Based on the anxious/careful behavior during semen collection, the similar clinical and histological findings compared with the remaining dogs, and the absence of spermatozoa in the ejaculate and urine, we postulated that dog no. 8 was suffering from NOA. Different to this, libido of dog no. 1 was excellent and behavior during semen collection “normal” why we postulate that low AlP activity and lack of spermatozoa in the ejaculate (and urine) were associated with obstruction. Interestingly, this dog was the only one not showing immune-cell infiltration associated with arrest of spermatogenesis, possibly confirming another etiology of infertility.

### Bacteriology and *B. canis* Serology

Bacteriological examination of semen male dogs suffering from azoospermia often reveals either sterile samples or detection of unspecific bacteria of the physiological flora (72, 73). Bacteria identified in the present samples, such as *Streptococcus canis, Staphylococcus pseudintermedius, Pasteurella multocida*, and *Moraxella* sp., had been previously described to be part of the physiological flora (17, 72–75). Furthermore, the number of CFUs ranged between 600 and 2,000 CFU/ml, confirming that clinically meaningful bacterial growth, defined by >10^5^ bacteria/ml (17, 75), was not found in any sample. The present findings are in good agreement with our earlier observation that neither the number of bacteria nor the bacterial species differ between dogs with normospermia, teratozoospermia, and azoospermia (73). However, as not only aerobic culture was performed in this but also the majority of earlier studies (17, 72–75), data do not allow for the final conclusion that samples were in fact sterile. So future studies should perform non-genomic sequencing on semen samples obtained from dogs with normal fertility and normospermia and various deviations in fertility and semen quality, including azoospermia, to identify all kinds of bacteria present in the respective samples.

Although the role of facultative bacteria for infertility/NOA in the dog is discussed controversially, there is no doubt about the obligate pathogenic potential of *B. canis*. *B. canis* manifests in reproductive failure, infertility in male and female dogs, late abortion, and autoimmune orchitis (AIO) (76). Canine brucellosis is reported worldwide. Diagnosis is by culture, serology, or polymerase chain reaction (PCR). As a culture of *B. canis* is challenging (77), PCR is the method of choice for direct identification (78–80). By now, *B. canis*-positive PCR was not reported from Denmark—where all animals had been residing—by now, with the total number of samples, however, being very limited (48). Different from this, a recent study retrospectively evaluating samples for agglutination test identified 5.4% (150/2,764) positive samples with 6/117 positive serum samples found in Denmark in total (48). Due to low accuracy of the available serologic methods, diagnosis remains very challenging, and brucellosis should always be considered as a possible cause for gonadal failure and reduced fertility. Nevertheless, antibodies against *B. canis* were not detectable by serum tube agglutination test in any of the 10 samples using a standardized method (48).

### Endocrine Analysis

A profound diagnostic evaluation of canine NOA should include the exclusion of endocrine disorders. In the current study, we included LH, testosterone, estradiol-17ß, and thyroidal analysis. Although of interest, FSH determination was not included in the present study due to the lack of functional canine tests. Even if some enzyme-linked immunosorbent assays were marketed for the dog, we were unable to produce repeatable, trustable data (own unpublished experiences).

Interpretation of hormonal data requires an awareness that hormone analysis is complex and that results can vary considerably depending on the method (81), making overall comparisons sometimes difficult or questionable. For this reason, in the current study, results obtained were mainly compared with results obtained with the same methods in the same laboratory. LH concentrations ranged within our normal reference interval between 0.5 and 4.0 ng/ml (44). In accordance with the present results, previous studies have demonstrated an elevated or normal LH concentration in cases of spermatogenic or gonadal failure in men and dogs (13, 71) and a decreased LH concentration in association with hypogonadotropic hypogonadism in men (71) and hypopituitarism in the male dog (17).

Concerning testosterone, serum concentrations were also in the range of normal intact dogs as described not only in our laboratory but also other studies [0.4–6.0 ng/ml, corresponding to 1.4–20.8 nmol/l: (49, 82)]. Whether a decreased peripheral testosterone concentration is an indicator of impaired or arrested spermatogenesis is discussed controversially. Previous studies investigating blood testosterone in azoospermic dogs identified concentrations within the normal reference interval (39–41), as well as significantly lower basal concentrations (42, 43, 45). As uncertainty arises from the discrepancy between the systemic circulation concentration and intratesticular testosterone level—which is maintained at ~100 times the level of the peripheral testosterone serum concentration [rodents: (83); men: (84, 85); boar: (86)], further studies should focus on the comparison of systemic and intratesticular testosterone concentrations in fertile and infertile dogs.

Mean physiological serum estradiol-17ß concentration in male dogs analyzed by the same radioimmunoassay was 96.5 ± 33.0 pmol/L [26.3 ± 9.0 pg/mL; (87)], which is partially higher than described elsewhere [7.34–74.52 pmol/L corresponding to 2.0–20.3 pg/ml; (49)]. This observation clearly confirms that evaluation requires taking specific methods and reference values into consideration. Based on the literature, estradiol-17ß concentrations measured in this study in azoospermic NOA dogs (66.0 to 141.5 pmol/L) would have been considered as high. They can, however, be considered within the normal range in relation to the laboratory-specific radioimmunoassay references. High estradiol concentrations (or high estradiol-17ß/ testosterone ratios) have been described as a useful biomarker for estrogen producing testicular pathology in various species [men: (88); dog: (89)]. Noteworthy, a possible tumorous cause of NOA had been ruled out by andrological and ultrasound examination. Besides, the histological assessment did not reveal any indication of a tumor pathology. Nevertheless, back in 1983, Winter et al. (90) demonstrated the significance of physiological estradiol concentrations through aromatization of testosterone for the control of gonadotropin secretion in the male dog. Although increased estradiol-17 ß concentrations had been described in two canine azoospermia patients (77 and 81 pmol/l) compared with two normospermic controls (29 and 45 pmol/l) (91), based on the laboratory specific references, we conclude that NOA was not associated with “hyperestrogenism” in our patients.

Investigations in various species demonstrated the impact of thyroidal dysfunctions, mainly hyperthyroidism and hypothyroidism, on reproductive functions and fertility. Although hyperthyroidism in men is associated with increased sperm abnormalities and significantly lower sperm motility (92) and affects sexual behavior (32), hypothyroidism is linked to decreased libido and impaired antioxidant defense mechanisms, resulting in reproductive dysfunction such as infertility (34). Regarding the male dog, the relationship between thyroid dysfunctions and reduced fertility or infertility is often predicted but rarely demonstrated. Although Johnston et al. (17) attributed infertility and a poor libido to hypothyroidism, Segalini et al. (38) found no difference in T4 plasma concentration between normo- and subfertile dogs. In addition, non-thyroidal illness, so-called Euthyroid sick syndrome, can affect and suppress thyroid hormones, making the diagnostic procedure even more complex. In the present study, one dog (no. 5) had decreased T4 and cTSH concentrations above the reference, indicating hypothyroidism, possibly indicating a link between thyroid disorders and azoospermia. Furthermore, thyroglobulin autoantibodies were above the reference levels in dog nos. 1 and 3. These results might indicate immune-mediated lymphocytic thyroiditis, as already described in a closed beagle colony (93). The familial incidence of lymphocytic thyroiditis is correlated with the occurrence of lymphocytic orchitis, which leads to focal degeneration, segmental and diffuse atrophy—just as seen in our histological evaluation. Likewise, autoimmune thyroiditis in men often combines with AIO or other autoimmune diseases (94). For both, thyroidal disorders and lymphocytic orchitis, heritability can be assumed (93) and should be evaluated in future research.

The detection of antisperm antibodies (ASAs) in the seminal plasma and/or on the sperm membranes of men or in fluids of the female genital tract is used as a diagnostic criterion for infertile couples (95). As an immunological response to spermatozoa, these antibodies bind to the sperm membrane and impair the sperms' ability to fertilize (96). In the dog, the presence of ASA (anti-sperm immunoglobulin G) was described in an AIO case report (97) but also temporarily subsequent to testicular biopsy (98). Interestingly, ASAs after testicular biopsy collections were not only transient but did not have any predictably negative effect on motility, too (98). Although ASAs were identified in bitches after immunization, and their presence was postulated to affect conception rates (99), the role of ASA in male dogs is poorly understood by now and requires further research. Nevertheless, ASA detection seems to be an interesting tool in the diagnostics of canine NOA patients. However, a commercially available, standardized test for the detection of autoantibodies against sperm in dogs is currently lacking.

### Histology

A key strength of the present study was the use of scissor biopsies to closer evaluate changes in testicular histology and morphometry. Although excisional wedge biopsies provide better samples for histological assessment compared with, *e.g*., fine-needle aspirates, it is often falsely accused of potential complications such as hemorrhage, infections, and a decrease in sperm production (41). Unsurprisingly, the fear of worsening the progression/infertility through testicular biopsies prevents many owners from assigning subsequent analyses. Recent studies have been able to confirm the safety of testicular biopsies as diagnostic procedures and could prove that there is no negative effect on total sperm output or motility (98). Additionally, our own clinical experiences and observations within the present study and beyond support these results, as no clinical adverse effects had been detected after biopsy sampling.

Previous studies have reported a failure of spermatogenesis and differentiation as one of the main histological characteristics of NOA in different species [men: (1, 71); dog: (19, 30, 41)], and evaluation of testicular biopsies indicated varying degrees of degeneration, germ-cell arrest, Sertoli-cell-only syndrome, or tubular atrophy (71, 100–104), also in the dog (17, 19, 30, 41, 60, 63). Similarly, we found slight to severe disruption of spermatogenesis in included canine NOA patients, including focal Sertoli-cell only, vacuolization, giant cells, multinucleated germ cells, and tubular shadows. Besides, we described for the first time a change in the composition of testicular compartments. The relative reduction of the tubular area is associated with a relative increase in interstitial and vascular areas. The reduction of the tubular area is related to the reduced diameter of the tubules and the disruption of spermatogenesis in NOA-affected dogs. This is similar to the earlier described reduction of the tubular compartment related to the slow release gonadotropin releasing hormone agonist implant treatment during downregulation of testicular endocrine and germinative function (57). Different from this, the increase of the interstitial area is explainable by the significant immune-cell infiltration and increase of connective tissues.

More surprisingly, however, we identified focal or generalized immune-cell infiltration in 9 of 10 dogs. The only dog not showing immune-cell infiltration was the one with suspected OA. These data underline that immune-cell infiltration plays an important role in infertility, namely NOA in the dog. Immune-cell infiltration had been described in dogs earlier and discussed as spontaneous AIO (17, 19, 30, 41, 60, 93, 97, 105). Spontaneous AIO was also described in minks (106, 107), mice (108, 109), and rats (110). Although some authors report spontaneous AIO in men (111–114), others question AIO in men and consider it as not established as a clinical entity (115). Even if earlier studies described AIO before, up to now, the relevance of AIO in canine NOA was only poorly understood (13, 19, 30, 63, 64, 93). Because 7 of 10 dogs in our study mated successfully before and had significant immune-cell infiltration into the testicular tissue, it might be speculated that AIO has to be considered as an important, if not *the* reason for acquired infertility in dogs. However, it remains questionable if the etiology of immune-cell infiltration is really spontaneous and primarily autoimmune, as postulated in earlier studies (17, 19, 30, 41, 60, 93, 97, 105). It seems more likely that immune-cell infiltration and subsequent irreversible breakdown of spermatogenesis are consequences of chronic orchitis of unknown origin with an asymptomatic course of the disease and unspecific clinical signs as previously described in man (115). It has even been discussed as a neglected cause of male infertility (115). The similarity with human testicular histopathology is striking, indicating that dogs suffering from acquired NOA associated with the described histopathology could be suitable models to study chronic orchitis, its development, and progression and might be helpful to develop specific therapeutic options.

## Conclusions

The results of our current investigation in infertile dogs do not only confirm that NOA occurs much more frequently than OA (9 of 10 patients affected) but also indicate that chronic degenerative changes associated with significant immune-cell infiltration are the cause for NOA in all nine infertile male dogs. As seven of nine dogs sired successfully before becoming infertile, we postulate that the histological findings with the lack of previous and current clinical signs of inflammation and disease indicate chronic asymptomatic immune-mediated orchitis. We further postulate that these findings have previously been interpreted as spontaneous AIO in dogs. Without a doubt, the current data support the role of testicular biopsies in infertility diagnostics in the dog. Nevertheless, follow-up studies should focus on the early identification of affected dogs, on uncovering underlying inflammatory processes behind spermatogenesis failure, and identifying possible treatment options to (re-)initialize spermatogenesis.

## Data Availability Statement

The raw data supporting the conclusions of this article will be made available by the authors, without undue reservation.

## Ethics Statement

Ethical review and approval was not required for the animal study because according to the respective Danish authorities (Dyreforsøgstilsynet Fødevarestyrelsen) no approval was necessary for sampling as sampling was for diagnostic purposes (clinical exam, semen collection, blood sampling and testicular biopsy sampling). Written informed consent was obtained from the owners for the participation of their animals in this study.

## Author Contributions

SG-P: conceptualization, sample collection, overall data analysis and preparation, statistical analysis, preparation of initial draft, review, and finalization of the manuscript. LR: histological evaluation, figure preparation, preparation of initial draft, review, and finalization of the manuscript. CB: histological evaluation, data analysis, statistical analysis, and preparation of the manuscript. GS: supervision of sex steroid determinations. A-KU: sample collection. HK: LH determinations, overall data analysis and preparation, statistical analysis, and preparation of the manuscript. All authors reviewed and approved the final manuscript.

## Funding

The authors gratefully thank the GkF (Society for Kynological Research, Bonn, Germany) for their financial support of the study. This Open Access publication was funded by the Deutsche Forschungsgemeinschaft (DFG, German Research Foundation) within the program LE 824/10-1 Open Access Publication Costs and University of Veterinary Medicine Hannover, Foundation.

## Conflict of Interest

The authors declare that the research was conducted in the absence of any commercial or financial relationships that could be construed as a potential conflict of interest.

## Publisher's Note

All claims expressed in this article are solely those of the authors and do not necessarily represent those of their affiliated organizations, or those of the publisher, the editors and the reviewers. Any product that may be evaluated in this article, or claim that may be made by its manufacturer, is not guaranteed or endorsed by the publisher.
